# Computational master-regulator search reveals mTOR and PI3K pathways responsible for low sensitivity of NCI-H292 and A427 lung cancer cell lines to cytotoxic action of p53 activator Nutlin-3

**DOI:** 10.1186/s12920-018-0330-5

**Published:** 2018-02-13

**Authors:** Ulyana Boyarskikh, Sergey Pintus, Nikita Mandrik, Daria Stelmashenko, Ilya Kiselev, Ivan Evshin, Ruslan Sharipov, Philip Stegmaier, Fedor Kolpakov, Maxim Filipenko, Alexander Kel

**Affiliations:** 1 Institute of Chemical Biology and Fundamental Medicine, SBRAN, Novosibirsk, Russia; 2 Biosoft.ru, Ltd, Novosibirsk, Russia; 3 geneXplain GmbH, D-38302 Wolfenbüttel, Germany

**Keywords:** Lung cancer cell lines, Sensitivity to anticancer drugs, Nutlin-3, p53, Transcription factors, TRANSFAC

## Abstract

**Background:**

Small molecule Nutlin-3 reactivates p53 in cancer cells by interacting with the complex between p53 and its repressor Mdm-2 and causing an increase in cancer cell apoptosis. Therefore, Nutlin-3 has potent anticancer properties. Clinical and experimental studies of Nutlin-3 showed that some cancer cells may lose sensitivity to this compound. Here we analyze possible mechanisms for insensitivity of cancer cells to Nutlin-3.

**Methods:**

We applied upstream analysis approach implemented in geneXplain platform (genexplain.com) using TRANSFAC® database of transcription factors and their binding sites in genome and using TRANSPATH® database of signal transduction network with associated software such as Match™ and Composite Module Analyst (CMA).

**Results:**

Using genome-wide gene expression profiling we compared several lung cancer cell lines and showed that expression programs executed in Nutlin-3 insensitive cell lines significantly differ from that of Nutlin-3 sensitive cell lines. Using artificial intelligence approach embed in CMA software, we identified a set of transcription factors cooperatively binding to the promoters of genes up-regulated in the Nutlin-3 insensitive cell lines. Graph analysis of signal transduction network upstream of these transcription factors allowed us to identify potential master-regulators responsible for maintaining such low sensitivity to Nutlin-3 with the most promising candidate mTOR, which acts in the context of activated PI3K pathway. These finding were validated experimentally using an array of chemical inhibitors.

**Conclusions:**

We showed that the Nutlin-3 insensitive cell lines are actually highly sensitive to the dual PI3K/mTOR inhibitor NVP-BEZ235, while no responding to either PI3K –specific LY294002 nor Bcl-XL specific 2,3-DCPE compounds.

**Electronic supplementary material:**

The online version of this article (10.1186/s12920-018-0330-5) contains supplementary material, which is available to authorized users.

## Background

One of the important targets in cancer cells is well known tumor suppressor protein p53. Among known candidate small molecules interacting with p53 and, therefore, counteracting proliferation of cancer cells is the cis-imidazoline analog compound called Nutlin-3 [1, 2]. Through interaction with the complex between p53 and its repressor Mdm2 the compound Nutlin-3 increases activity of p53, which, in turn, leads to an increase in the apoptosis of the cancer cells. Clinical and experimental studies of Nutlin-3 and other Mdm2/p53 binding inhibitors showed that some cancer cells are not sensitive to these compounds. The dominant mechanism of resistance to such inhibitors is the mutant status of *TP53* (gene encoding p53 proteins) (https://www.ncbi.nlm.nih.gov/pubmed/25730903). There is nevertheless a wide range of sensitivity to the Mdm2/p53 binding inhibitors among *TP53* wild-type cancer cell lines, which vary widely for different inhibitors (which in turn clearly emphasizes differences of the particular molecular mechanisms of action of different Mdm2-p53 inhibitors) [3]. One of the possible mechanisms of the relative insensitivity to these inhibitors (including Nutlin-3) of such cell lines is a high activity of one or more pro-survival pathways precluding insensitive cells from entering apoptosis even in presence of the cytotoxic compound. Such highly active pro-survival pathways can be either present in the cancer cells ab-initio (due to some “favorite” expression pattern of respective components of the signaling pathways), or such pro-survival pathways are activated in the cancer cells during and sometime as a result of the treatment using various chromatin reprogramming mechanisms [3]. In this work we focus our attention on the pro-survival pathways that are present and active ab-initio in some of lung cancer cell lines that are relatively insensitive to the p53 re-activating compound Nutlin-3. Detection of such pre-existing pathways in the populations of cancer cells can help in selecting appropriate drug treatment that either kill the cancer cells along or potentiate the response to Mdm2/p53 binding inhibitors as it is demonstrated previously for various cancer cell lines [4].

Experimental identification of activated pathways and corresponding potential drug targets in cancer cells is time consuming and very expensive. Computational analysis of gene expression data can help to identify few candidate pathways that can be validated experimentally in focused experiments. Many of such gene expression data are deposited in databases such as ArrayExpress [5] or Gene Expression Omnibus (GEO) [6], and can be used in combination with own gene expression data to identify expression signatures specific for particular cell types and cellular conditions. Such signatures can be used directly for selection of potential drug targets using the mere statistical significance of the expression changes. For a more refined analysis of the molecular mechanisms a conventional approach of mapping the differentially expressed gene (DEG) sets to Gene Ontology (GO) categories or to KEGG pathways, for instance by GSEA (gene set enrichment analysis), is usually applied [7, 8].

But, such approaches provide only a very limited clue to the causes of the observed phenomena and therefore not very useful for selection of potential drug targets. To overcome such limitations we introduced earlier a novel strategy, the “upstream analysis” approach for causal interpretation of the gene expression signatures and identification of potential master regulators [9–13]. This strategy comprises two major steps: (1) analysis of promoters of genes in the signatures to identify transcription factors (TFs) involved in the process under study (done with the help of the TRANSFAC® database [14] and site identification algorithms, Match [15] and CMA [16]); (2) reconstruction of signaling pathways that activate these TFs and identification of master-regulators on the top of such pathways (done with the help of the TRANSPATH® signaling pathway database [17] and special graph search algorithms implemented in the geneXplain platform [12]). In this paper we applied our upstream analysis algorithm to identify master regulators potentially responsible for dumping down the sensitivity of particular lung cancer cell lines to the cytotoxic activity of p53 reactivating compound Nutlin-3.

Many tumor cells are characterized by a substantial increased expression of p53 inhibitor Mdm2 [18]. In these cells p53 is rapidly degraded allowing an escape from p53-dependent apoptosis. The destruction of the Mdm2-p53 complex stabilizes the pool of p53 and the restores its activity, which, in turn, leads to inhibition of proliferation and / or death of tumor cells. To date, three classes of small molecular inhibitors of Mdm2-p53 interaction are identified, namely, Nutlins (nutlins) [19], BDAs (benzodiazepindiones) [20] and a series of spiro-oxindole derivatives MI-63, MI-219 and MI-43 [21, 22]. All three series of compounds bind with high affinity to p53-specific pocket region of Mdm2, thus, displacing p53 from its complex with Mdm2.

Among these compounds, Nutlin-3 is the most commonly used in the anti-cancer studies. Pre-clinical trial data of Nutlin-3 for the treatment of acute myeloid leukemia [23, 24] has confirmed its ability to induce apoptosis of tumor cells, while sparing normal hematopoietic cells. During last years, the small molecule drug RG7112, a derivative compound of Nutlin-3, was studied in several Phase I clinical trials for advanced solid and hematological cancers, and for liposarcoma [1] (clinical trial: NCT00559533), for acute myelogenous leukemia (clinical trial: NCT01635296) and for soft tissue sarcoma (clinical trial NCT01605526), delivering promising results [2]. In those studies, the cases of the resistance and low sensitivity of cancer cells to the treatment by Nutlin-3 were noted. Among most recent and most promising Mdm2/p53 binding inhibitors that are in clinical trials now we can mention AMG-232 (https://clinicaltrials.gov/ct2/show/NCT03041688) and HDM201 (https://clinicaltrials.gov/ct2/show/NCT02343172). No gene expression data on these two compounds are available in GEO yet. We hope that our current study of Nutlin-3 insensitivity mechanisms will help to prepare the background for future studies of such more potent compounds as AMG-232 and HDM201.

In order to analyze the effect of Nutlin-3 on lung cancer cells and understand the factors rendering these cells sensitive or insensitive to the treatment we performed a focused study of its biological activity in various lung cancer cell lines carrying wild-type TP53 gene as well as TP53 inactivating mutations. A total of 8 cell lines were analyzed: A549, NCI-H292, A427, COR-L23, DV-90, NCI-H1395, NCI-H1944, NCI-H2228. The cytotoxic activity of Nutlin-3 on lung cancer cell lines was determined based on the IC_50_ (inhibitory concentration) parameter (concentration of compound leading to 50% of the cell death in the culture). The IC_50_ parameter was determined using a resazurin viability test. We found that lung cancer cell lines are significantly differing from each other in respect to the sensitivity to Nutlin-3.

Based on our drug sensitivity measurements, we chose one of the most Nutlin-3 sensitive cell lines - H1944, and two less sensitive (we call them as insenistive) cell lines - A427 and NCI-H292 for further studies. We performed microarray experiments on these cell lines before and after treatment by Nutlin-3 in order to reveal different gene expression profiles in sensitive and insensitive cell lines. Comparative analysis of microarray data of these three cell lines was done using a computational pipeline “From genome to target” (http://my-genome-enhancer.com) implemented using BioUML driven systems biology platform (www.biouml.org, geneXplain platform: www.genexplain.com). We revealed a number of differentially expressed genes (DEGs) between sensitive and insensitive cell lines. Promoter and pathway analysis of these DEGs using “upstream analysis” approach [13] helped us to identify potential master regulators in these cancer cell lines responsible for the elevated resistance to Nutlin-3. Among them, the most promising was mTOR as one of the most important regulator of pro-survival mechanisms in the cells. We applied specific chemical inhibitors in order to test their effect on these cell lines. We found that used Nutlin-3 insensitive cell lines exhibit the highest sensitivity to the dual chemical inhibitor of mTOR-PI3K whereas the Nutlin-3 sensitive cell line appeared to be relatively insensitive to this inhibitor. These results confirmed our prediction of the master regulators in the mTOR-PI3K signaling pathway responsible for the elevated resistance of particular lung cancer cell lines to treatment by the p53-reactivating compound Nutlin-3. As we predicted, the Nutlin-3 insensitive cell lines appeared to be highly sensitive to the inhibitors of mTOR-PI3K pathway. These findings open a promising possibility for a combinatory therapy combining Nutlin-3 with mTOR-PI3K inhibitors. Such drug combinations will have a potential to tackle the observed heterogeneity between different cancer cell linages towards p53-reactivators such as Nutlin-3.

## Methods

### Cell lines

In our study we used the following eight lung cancer cell lines, seven of them are classical non-small cell lung cancer (NSCLC) cell lines (A549, A427, COR-L23, DV-90, NCI-H1395, NCI-H1944, NCI-H2228) and one is cell line of the mucoepidermal lung carcinoma (NCI-H292), which is also classified as NSCLC according to the existing classification (https://radiopaedia.org/articles/mucoepidermoid-carcinoma-of-lung). The characteristics of these eight cell lines are given in the Table 1.

**Table 1:** NSCLC cell lines used in this work

Cell line	Type of cancer	P53 status
A427	NSCLC adenocarcinoma	wt
A549	NSCLC adenocarcinoma	wt
COR-L23	NSCLC large cell adenocarcinoma	P53 inactivating alteration
DV-90	NSCLC adenocarcinoma	wt
NCI-H1395	NSCLC adenocarcinoma	wt
NCI-H1944	NSCLC adenocarcinoma	wt
NCI-H2228	NSCLC adenocarcinoma	P53 inactivating alteration
NCI-H292	Mucoepidermal lung carcinoma	wt

### Conditions of cultivation

The cells were cultured in DMEM / F12 medium containing 10% FBS, 0.03% glutamine, and kanamycin at a concentration of 100 μg / ml at 37 °C and 5% CO2 in the atmosphere. When the cell culture reached a density of ~ 70%, the monolayer cells were seeded with 1: 4 culture dilution. The described cultivation conditions allowed maintaining the cell culture in the exponential growth.

### Test of sensitivity of lung cancer cell lines to Nutlin-3

Cells from frozen cultures were seeded in 25 cm2-culture flasks. Before testing the compound, the cells must be reseeded at least once. To test the sensitivity to the compound treatment, the lung cancer cells in the exponential growth phase were seeded in wells of a 24-well plate at 60,000 cells per well. After 24 h, the culture medium was replaced with a fresh one containing the compound in the following concentrations: Nutlin-3 (Nutlin3 (±), Cayman Chemical) at concentrations of 34 μM, 17 μM, 8.5 μM, 4.25 μM, 2.2 μM and 0 μM (control).

Each concentration point was represented in triplicates. 48 h after the addition of the compound the proportion of viable cells was determined by a viability test with resazurin.

### Test for vitality with resazurin

Resazurin is an oxidation-reduction dye and is used to analyze the toxicity of compounds in cell culture [25]. The analysis is based on the ability of metabolically active cells to restore resazurin (a blue product) to resorufin (a pink color product, a fluorophore with absorption and emission maxima at 571 and 586 nm, respectively). The conversion of resazurin to resorufin is carried out intracellularly and is provided by mitochondrial, microsomal and cytosolic oxidoreductases. Thus, the amount of resorufin in the culture medium is related to the total number and (or) survival of the cells in the culture.

The viability test was performed 48 h after the cell culture was incubated with the test compound. To do this, the culture medium with the test compound was removed from the wells of the plate and 500 μl of Dulbecco’s solution (1 ×) containing rezazurin (resazurin sodium salt, Diaam) at a concentration of 50 μg / ml was added. A 500 μl Dulbecco solution with resazurin (50 μg / ml) added to an empty well of a 24-well culture plate was used as control (background). The time of incubation of cells with resazurin was 1 h. The incubation was carried out under sterile conditions, at 99% humidity, at 37 °C and at a concentration of 5%. Accumulation of resorufin (reduced form of resazurin) was assessed by fluorescence detection in the range of 587/607 nm. To assess the results of the test, a calibration curve was constructed for the change in fluorescence as a function of the number of cells. Using this calibration curve we determined the relationship between the intensity of fluorescence and the number of viable cells in culture. The proportion of surviving cells was used to construct the dose-effect curve for each cell line. The IC_50_ value was calculated from these curves obtained using the Probit Analysis 1.0 software [26].

### Isolation of cellular RNA and synthesis of cDNA

The cells of the lung cancer lines were lysed with Trisol reagent (Invitrogen). The total RNA was isolated according to the recommendations of Invitrogen. The quality of RNA preparations was assessed by electrophoretic separation in a 1.3% agarose gel.

The reverse transcription reaction was performed at 42 °C for 45 min in 20 μl of a reaction mixture containing 10 mM Tris-HCl (pH 8.3), 5 mM MgCl2, 10 mM DTT, 50 mM KCl, 0.2 mM dNTP, Stat-9 primer (10 ng / Μl), 100 unit act., DNA-dependent RNA polymerase MoMLV (Biosan, ICBPM SORR) and 500 ng of the total RNA.

Amplification reactions were performed using thermal cyclers with an optical unit for detecting the fluorescence of iQ5 iCycler or CFX96 (Bio-Rad). The possibility of recording fluorescence in real time was achieved by adding to the reaction mixture the intercalating dye SYBRGreen I.

#### Microarray analysis

Microarray analysis was done using the following microarray platform: Human HT-12 v3 Expression BeadChips (Ilumina). Three cell lines: А427, H292 and H1944 were treated by Nutlin-3 in a concentration that maximally discriminates the sensitive and moderately insensitive cell lines (5 μM) and also in the maximal cytotoxic concentration (30 μM). The cells were incubated with the compound during 24 h. The experiment was done in two biological replicates for each condition. The raw data files with measured gene expression are deposited in the GEO (https://www.ncbi.nlm.nih.gov/geo/). In order to detect differentially expressed genes the raw microarray data were normalized and further analyzed using Limma tools from the R/Bioconductor package integrated into the BioUML/geneXplain driven pipeline “From genome to target”. The Limma has calculated LogFC between mean expression values of the genes in two insensitive cell lines in comparison with the sensitive cell line (the logarithm on the basis of 2 of the fold change between different conditions) and the *p*-value and adjusted p-value (corrected to the multiple testing). In order to take into account the correlated nature of technical replicates (duplicates), which were done for each of the cell lines, we applied the “block” option in the Limma analysis in order to compute the *p*-values with better precision. (see the respective R script in the Additional file 1: Figure S1). All these parameters were used to detect differentially expressed genes.

#### Analysis of enriched transcription factor binding sites

Transcription factor binding sites in promoters of differentially expressed genes were analyzed using known DNA-binding motifs described in the TRANSFAC® library [14], release 2017.2 (geneXplain, Wolfenbüttel, Germany) (http://genexplain.com/transfac). The motifs are specified using position weight matrices (PWMs) that assign weights to each nucleotide in each position of the DNA binding motif for a transcription factor or a group of them.

The pipeline “From genome to target” provides tools to identify transcription factor binding sites (TFBS) that are enriched in the promoter regions under study as compared to a background sequence set such as promoters of genes that were not differentially regulated under the condition of the experiment. We denote study and background sets briefly as Yes and No sets. The algorithm for TFBS enrichment analysis, called F-Match, has been initially described in [15]. Briefly, as it has been described in detail previously [13], the procedure finds a critical value (a threshold) for the score of each PWM in the library that maximizes the Yes/No ratio *R*_*YN*_ as defined in Eq. (1) under the constraint of statistical significance.

1 $$ {R}_{YN}=\frac{Sites_{Yes}/{Sites}_{No}}{Seq_{Yes}/{Seq}_{No}} $$

In Eq. (1), *Sites* and *Seq* are the sites and sequences counted in Yes and No sets. High Yes/No ratio indicates strong enrichment of binding sites for a given PWM in the Yes sequences. The statistical significance is computed as follows:

2 $$ {\displaystyle \begin{array}{c}P\left(X\ge x\right)=\sum \limits_{n=x}^N\left(\genfrac{}{}{0pt}{}{N}{n}\right)\bullet {p}^n\bullet {\left(1-p\right)}^{N-n}\\ {}p=\frac{Seq}{Seq_{Yes}+{Seq}_{No}}\\ {}n={Sites}_{Yes};N={Sites}_{Yes}+{Sites}_{No}\end{array}} $$

This binding site enrichment analysis is carried out in the Site Analysis module of the pipeline “From genome to target”. We consider for further analysis only those TFBSs that achieved a Yes/No ratio > 1 and a *P*-value < 0.01. The pipeline further maps the matrices to respective transcription factors, and generates visualizations of all results.

#### Finding master regulators in networks

We searched for master regulator molecules in signal transduction pathways upstream of the identified transcription factors using appropriate tools of the pipeline “From genome to target”. The master-regulator search uses the TRANSPATH® database (http://genexplain.com/transpath) [17]. A comprehensive signal transduction network of human cells is built by the network analysis module of the pipeline using reactions annotated in TRANSPATH®. The main algorithm of the master regulator search has been described earlier [13]. The goal of the algorithm is to find nodes in the global signal transduction network that may potentially regulate the activity of the set of transcription factors found at the previous step of analysis. Such nodes are considered as most potent drug targets, since any influence on such a node may switch the transcriptional programs of hundreds of genes that are regulated by the respective TFs. In our analysis we have run the algorithm with a maximum radius of 12 steps upstream of the TFs.

#### Computational pipeline «from genome to target»

Comparative analysis of microarray data of these three cell lines was done using a computational pipeline “From genome to target” (http://my-genome-enhancer.com) implemented using BioUML driven systems biology platform (www.biouml.org, www.genexplain.com). The pipeline is capable of taking various multi-omics data (such as Genomics, Transcriptomics, Epigenomics, Proteomics and Metabolomics) and automatically performing the “Upstream analysis” [13] to detect master regulators as potential drug targets. It provides a flexible graphical tool for description of meta-data of the experiment that helps researcher to define data for the automatic analysis. The pipeline consists of four modules: Statistics, Genome Enhancer, Drugs, Targets. Depending on the input data, meta-data and the chosen parameters, the system generates a tailor-made data analysis workflow going through all these four modules. At the first step, data are statistically analyzed, and the lists of differentially expressed genes (DEGs) (in case of Transcriptomics data), proteins (Proteomics) and metabolites (Metabolomics) are prepared for the next steps of analysis. In case of Epigenomics data (ChiP-seq, DNA methylation), a list of statistically significant peaks and CpG methylation sites is prepared for the next step. The Genomics data are also analyzed at this step, and lists of revealed mutations are computed in the form of VCF files. At Genome Enhancer module the analysis of gene regulatory regions of differentially expressed genes is performed using Match™ [15] and CMA [16] tools (using TRANSFAC® [15], HOCOMOCO [27] and GTRD [28] databases of position weight matrices) in order to detect transcription factor binding sites (TFBSs) in the promoters and enhancers of DEGs. When available, ChIP-seq peaks and CpG methylation sites are used in this module to help to define the enhancer and silencer regions of the differentially expressed genes. In addition, mutations found inside enhancers and silencers were used to extract transcription factor binding sites significantly affected by these mutations. As a result, a list of transcription factors regulating genes through the identified TFBSs is forwarded to the network analysis in the signal transduction network (using TRANSPATH®, REACTOME, HumanCyc databases). If genomic data is available, then the observed mutations in the coding regions of proteins, which are involved in signal transduction and which damage the function of these proteins, are taken into account. The network is then modified by exclusion of corresponding nodes and reactions from it. The network analysis algorithm reveals master regulators as it is described above. In pipeline modules “Drugs” and “Targets” the revealed master-regulators are interrogated and prioritized in order to select the most promising therapeutic targets. Various properties of these mater-regulator proteins, such as potential “drugability” (possibility to find known drugs or novel chemical compounds potentially interacting with these proteins) as well as additional annotation from HumanPSD® (www.genexplain.com/humanpsd) database about known disease relevance of these proteins, are taken into account for such prioritization. Finally, the pipeline “From genome to target” reports a short list of most promising targets.

## Results

### Sensitivity of different lung cancer cell lines to Nutlin-3

To test the sensitivity to Nutlin-3 we treated the selected lung cancer cell lines during 24 h by the compound in the following concentrations: 34 μM, 17 μM, 8.5 μM, 4.25 μM, 2.2 μM and 0 μM (control).

With the help of the viability test with resazurin we constructed the curves of the percentages of survived cells under increasing concentration of Nutlin-3 and identified IC50 values of Nutlin-3 in the eight lung cancer cell lines (A549, NCI-H292, A427, COR-L23, DV-90, NCI-H1395, NCI-H1944, NCI-H2228). We obtained the following results.

**Fig. 1:**
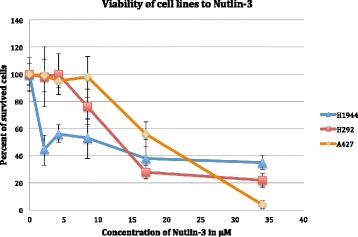
Viability test of three NSCLC cell lines with increasing concentration of Nutlin-3. Cell line H1944 shows higher sensitivity to the compound compared to the cell lines H292 and A427

The H1944 line was the most sensitive to the action of Nutlin-3 (IC_50_ = 4.9 ± 7.6). The other cell lines showed very different cytotoxic effect of Nutlin-3: H1395 (IC_50_ = 11.4 ± 3.8), DV-90 (IC_50_ = 12.9 ± 7.6), COR-L23 (IC_50_ = 15.1 ± 4.6), H292 (IC_50_ = 15.4 ± 2.6), A427 (IC_50_ = 18.8 ± 3.4). The A549 and H2228 cell lines were quite resistant to Nutlin-3, IC_50_ values were 31.2 ± 5.2 and 33.1 ± 6.7, respectively. Interestingly, that the cell line H2228 which carry the p53 inactivating mutation indeed has demonstrated the highest resistance to Nutlin-3. For the further analysis we selected three cell lines: one, which was the most sensitive to Nutlin-3 – the H1944 cell line, and two, which were still reacting to Nutlin-3, but only under relatively high concentrations – H292 and A427 (moderately insensitive). In the Fig. 1 one can see the clear differences in the cell survival dose-effect curve between the sensitive cell line and these moderately resistant once.

### Microarray experiments on Nutlin-3 sensitive and insensitive lung cancer cell lines

In order to get a comprehensive gene expression profile of the studied cell lines before and after treatment by Nutlin-3 we applied Illumina microarrays (Human HT-12 v3 Expression BeadChips). We treated three cell lines: А427, H292 and H1944 by Nutlin-3 in a concentration which maximally discriminates the sensitive and insensitive cell lines (5 μM) and in the maximally cytotoxic concentration (30 μM) (so high that it is potentially already off-target) that gives the end point in the dose-effect curve where no differences in survival between all cell lines were observed,. After data normalization we applied Limma tools (R/Bioconductor package integrated into the pipeline “From genome to target”) and compared gene expression in the insensitive cell lines (A427 and H292) with gene expression in the sensitive cell line (H1944) before treatment and after treatment by two concentration of Nutlin-3. Limma has calculated LogFC (the logarithm on the basis of 2 of the fold change between different conditions), the *p*-value and the adjusted p-value (corrected to the multiple testing) of the observed fold change In the Additional file 2: Table S1 we provide the normalized expression values of all genes with detected expression in the studies conditions and mapped to Ensembl.

### Dynamic gene expression changes upon treatment by Nutlin-3 of sensitive and insensitive lung cancer cell lines

In order to study the behavior of genes in the insensitive and in the sensitive cell lines after treatment of these cells by 5 μM and 30 μM of Nutlin-3 we computed the LogFC for the change of gene expression in these cell lines before and after the treatment. In Table 2 one can see the results of detection of Up- and Down- regulated genes upon treatment by Nutlin-3 in two concentrations (p-value< 0.05, LogFC> 0.58 (which corresponds to FC > 1.5) for up-regulated and LogFC<− 0.58 for down-regulated genes).

**Table 2:** Genes differentially expressed (UP and DOWN) upon treatment by Nutlin-3 in insensitive and in sensitive cell lines

Nutlin-3 Dosage (μM)	Sensitive		Insensitive	
	UP	DOWN	UP	DOWN
5	8	0	28	5
30	34	33	147	127

GO analysis of these differentially expressed genes clearly confirms the well known molecular mechanism of action of Nutlin-3. In the Additional file 3: Table S2 we summarize all results of GO analysis of these 7 sets of genes. Results of this analysis show high similarity of the processes triggered by Nutlin-3 treatment in all studied cell lines. Such processes as “cellular response to stress”, “cell cycle arrest”, “cell death” and “apoptotic process” are clearly most up-regulated in these cells upon Nutlin-3 treatment, and the processes, such as: “DNA replication initiation”, “S phase”, “cell cycle” and “DNA repair” are down-regulated. This reflects the major effect of Nutlin-3 on cancer cell as an activator of cell cycle arrest and apoptosis through inhibition of Mdm2-p53 complex.

**Fig. 2:**
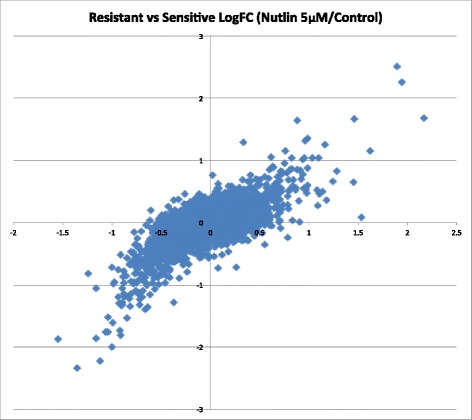
Plot comparing LogFC of gene expression changes upon treatment by 5 μM of Nutlin-3 in comparison to Control cells in the insensitive cell lines (x axes) versus the sensitive cell line (y axes)

In the plot bellow (Fig. 2) we compared the LogFC calculated in the insensitive cell lines and LogFC in the sensitive cell line. One can see a very good and highly statistically significant correlation between these two values (*r* = 0.69).

We also did the same comparison for the cells treated by 30 μM of Nutlin-3 (data not shown). We detected even higher similarity between gene changes in the sensitive and the insensitive cell lines (*r* = 0.776).

**Fig. 3:**
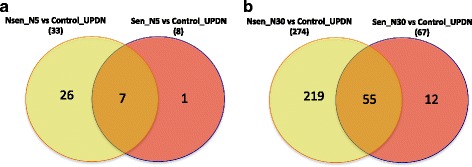
Venn diagrams of the differentially expressed genes upon treatment by two different dosages: (**a**) 5 μM and (**b**) 30 μM. We compare genes up- and down-regulated in the insensitive cell lines (yellow) versus sensitive cell line (pink)

Still, in order to reveal genes that differently changed their expression between insensitive and sensitive cell lines we applied filtering of the genes that statistically significantly changed their expression in opposite direction or genes that significantly changed expression in one type of cell lines but were non-changed in the other type. In both comparisons, upon treatment by 5 μM and 30 μM of Nutlin-3 no genes were found that would change their expression in opposite directions. Upon treatment by 5 μM of Nutlin-3 very few genes were found to be differentially expressed, and among them only 26 genes that statistically significantly changed their expression (21 up and 5 down) in insensitive cell lines upon treatment by Nutlin-3 and did not change their expression in the sensitive cell line. (Figure 3). The most statistically significant GO group with these up-regulated genes appeared to be “cell death” (*n* = 5, *p* < 2.7*10^− 3^). Namely the following genes important for this biological process: CDIP1, DPYSL4, DRAM1, TNFRSF10B, TP53INP1 found to be upregulated in the insensitive cell lines and not in the sensitive cell lines. We identified 219 genes that statistically significantly changed their expression (120 up and 99 down) in the insensitive cell lines upon treatment by 30 μM of Nutlin-3 and did not change their expression in the sensitive cell line. (Figure 3). It was interesting to see that for these genes we found enriched the following GO terms: for up-regulated genes “cellular amino acid metabolic process” (*n* = 9, *p* < 2.4*10^− 4^), “response to endoplasmic reticulum stress” (*n* = 6, *p* < 6.4*10^− 4^) and for down-regulated genes - “cell cycle process” (*n* = 22, *p* < 6.4*10^− 9^), “organic cyclic compound binding” (*n* = 54, *p* < 3.5*10^− 7^) and “nucleic acid metabolic process” (*n* = 42, *p* < 1.7*10^− 7^). This analysis shows that several genes that belong to these pathways have got different dynamics of the change of their expression upon treatment by Nutlin-3 in sensitive and in-sensitive cell lines. These differences can be potentially used as promising “dynamic” biomarkers of the sensitivity of the cancer cells to the treatment by Nutlin-3. Still, the causative mechanism of these differences remains rather unclear.

### Static gene expression profile differences between sensitive and insensitive lung cancer cell lines

Here, we performed a direct comparison of gene expression profile between Nutlin-3 sensitive and insensitive cell lines ab-initio (before the treatment) and after treatment by Nutlin-3 (in two concentrations). Such static comparison of the gene expression profiles can help us to detect stably active pro-survival pathways in these cells that protect these cells from cytotoxic activity of Nutlin-3.

In Table 3 one can see the results of detection of genes with increased and decreased expression in insensitive cell lines compared to the sensitive cell line in all three conditions (*p*-value< 0.05, LogFC> 0.58 for genes with increased expression and LogFC<− 0.58 for the genes with decreased expression).

**Table 3:** Table 3. Genes differentially expressed (Increased and Decreased) in Nutlin-3 insensitive cell lines as compared to the sensitive cell lines at baseline and under increasing dosage of Nutlin-3 stimulation.

Nutlin-3 Dosage (μM)	Increased	Decreased	Total
0 (Control)	257	366	623
5	297	377	674
30	258	368	626

One can see that the number of differently expressed genes between sensitive and insensitive cell lines is rather stable in all three conditions.

**Fig. 4:**
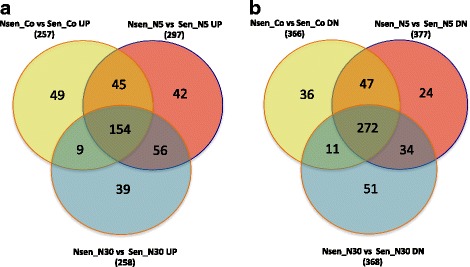
Venn diagrams of the differentially expressed genes of Nutlin-3 moderately insensitive cell lines versus sensitive cell lines after treatment by Nutlin-3 in two different dosages (5 μM and 30 μM) and in control. **a** Genes with increased expression; **b** Genes with decreased expression. Full LogFC computations for all three conditions can be found in Additional file 2: Table S1 (tab Nsen vs Sen)

Comparison between revealed deferentially expressed genes in these three conditions is shown by Venn diagrams (Fig. 2). One can see a significant overlap between DEG lists in all three conditions showing that the differences in cell line types determine quite a big portion of the gene expression profile. These genes show clear differences in their expression independently of the treatment conditions. Among the 154 genes with increased expression in the insensitive cell lines that are common in all three conditions (Fig. 4) we can see the enrichment of such GO terms as: “nucleotide-excision repair, DNA damage removal” (*n* = 4, *p* < 1.7*10^− 5^), “metabolic process” (*n* = 99, *p* < 7.9*10^− 5^) and “cell death” (*n* = 15, *p* < 4.6*10^− 3^). Among the 272 common genes with decreased expression some more specific GO terms showed up, such as: “oxidation-reduction process” (*n* = 38, *p* < 1.9*10^− 8^), “lipid metabolic process” (*n* = 40, *p* < 4.7*10^− 8^) and, most interestingly, “positive regulation of cell death” (*n* = 26, *p* < 1.9*10^− 8^). In the last category, among the genes with most decreased expression in the insensitive cell lines, we can mention such important genes as, S100A9, MLLT11, OSGIN1, CCL5, DAPK1, TNFRSF1A, SMAD3, LYN. Most probably, the low expression of these genes that are able to positively regulate the cell death process promotes the decreased sensitivity of the respective cell lines to the cytotoxic activity of Nutlin-3.

**Fig. 5:**
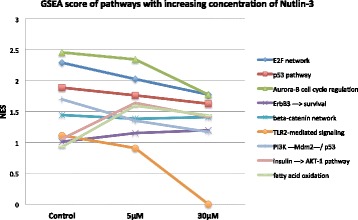
Graphic of the change of GSEA enrichment score NES of some important pathways in the insensitive cell lines as compared to sensitive cell line in different conditions with increased dosage of treatment by Nutlin-3

We performed gene set enrichment analysis (GSEA) of the obtained three gene expression profiles of differences between sensitive and insensitive lung cancer cell lines. For that we used geneXplain platform and applied the pathways ontology of TRANSPATH® database. The most enriched pathways are presented in the Additional file 4: Table S3.1 and S3.2 as well as in Fig. 5.

In the Fig. 5 we selected several TRANSPATH® pathways that demonstrated an interesting behavior of the GSEA enrichment value (NES) in insensitive cell lines as compared to the sensitive cell line in different conditions with increased dosage of treatment by Nutlin-3. One can see that such pathway as “beta-catenin network” is relatively stable in the gene enrichment, which demonstrates that genes belonging to this pathway have shown different expression in the insensitive and sensitive cell lines independently on the stimuli. Such pathways as E2F network, p53 pathway, PI3K pathway, Aurora-B cell cycle regulation and TLR2-mediated signaling showed a decrease of enrichment value in the cells under treatment by Nutlin-3. Interesting to see that such pathways as “insulin - > AKT-1 pathway” and “fatty acid oxidation” demonstrated an increase in enrichment value under treatment by Nutlin-3. The observed difference between insensitive and sensitive cell lines in expression of the genes that belong to these pathways appeared to be quite dependent on the treatment by Nutlin-3. Generally we observed more enriched pathways in the cells before Nutlin-3 treatment then after such treatment (see Additional file 4: Table S3.1).

**Fig. 6:**
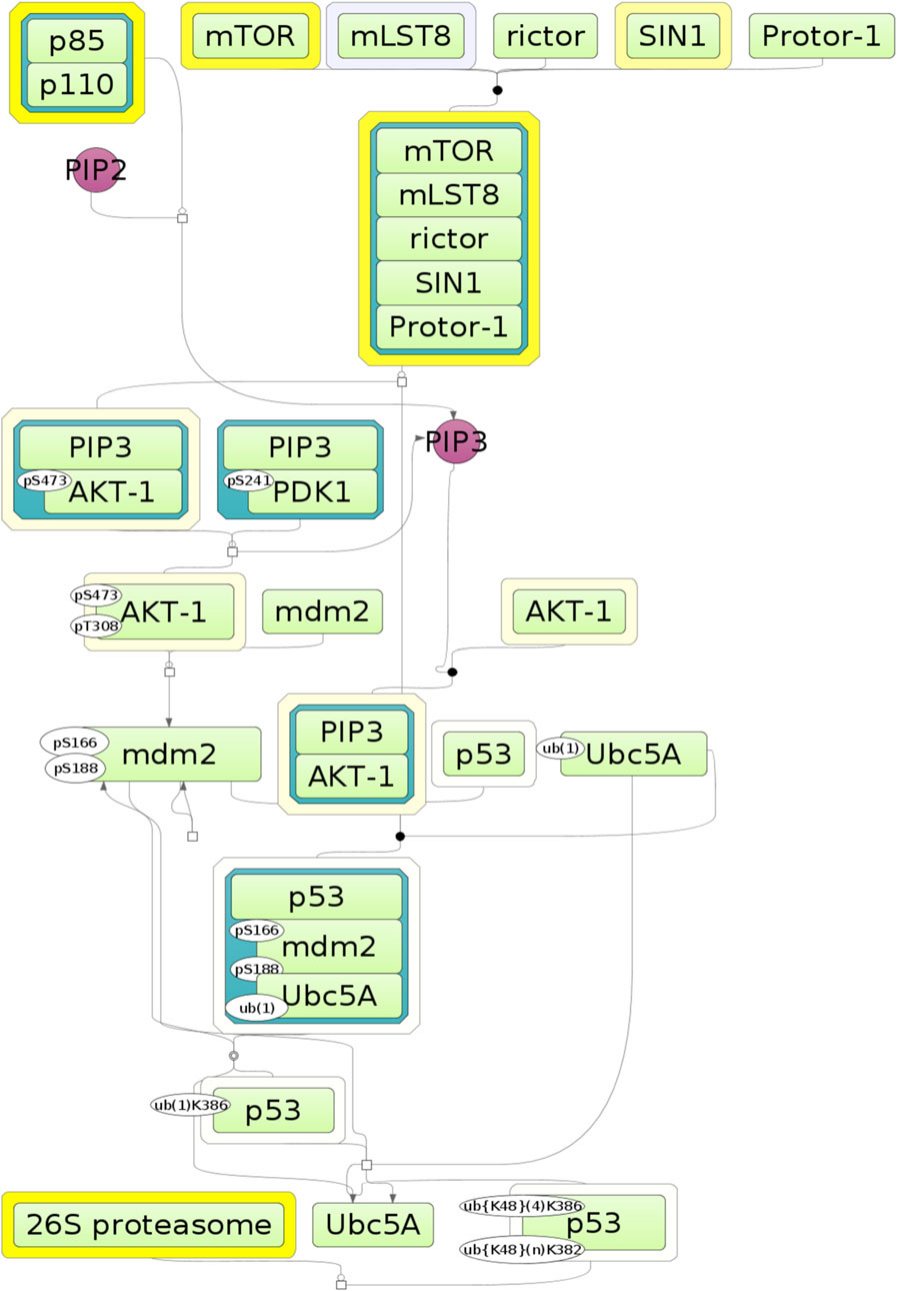
Visualization of canonical PI3K sub-pathway that leads to inhibition of p53 (PI3K ---Mdm2-−−/ p53). PI3K is represented on the diagram as the complex “p85:p110”. Yellow shadow around molecules represents the LogFC of the gene expression differences between Nutlin-3 insensitive and sensitive cell lines

In the Additional file 4: Table S3.2 we show the most representative (with group size higher then 30) and enriched pathways for the pre-treatment phase sorted according to the enrichment score NES. It is interesting to see that the most enriched pathways are represented by pathways involved in regulation of cell cycle, such as “Metaphase to Anaphase transition”, “cyclosome regulation”, “Aurora A and B regulation” and in general the networks of E2F and p53 transcription factors. Next pathway in the list that attracts our attention was PI3K sub-pathway (PI3K ---Mdm2-−−/ p53), with the genes that are quite different from the cell cycle related pathways (see Additional file 1: Figure S2). In the Fig. 6 bellow we show the PI3K sub-pathway with the Nutlin-3 sensitivity related log-fold change values of the expression of the genes encoding the components of this pathway.

From the “dynamic” and “static” analysis results we may conclude that although we can detect a number of genes that play their definite role in the “dynamic” creation of cell insensitivity to the drug after the treatment, still the major differences in gene expression of hundreds of genes in these cell lines is observed in “static” comparisons of these cell lines. Moreover, in the “static” analysis the “pre-treatment” comparison of the cell lines delivers the maximum number of enriched pathway. Therefore, in the following analysis we pay our main attention to the genes that we found differentially expressed between insensitive and sensitive cell lines before their treatment by Nutlin-3. We think, that such pre-treatment analysis is most promising in terms of clinical applications, since it can be done on the tumor samples before any treatment and can be used for further definition of the proper anti-cancer therapies.

### Enriched transcription factor binding sites in promoters of Nutlin-3 moderately resistant cell lines

Transcription factor binding sites in promoters of differentially expressed genes were analyzed using known DNA-binding motifs described by PWMs in the TRANSFAC® library, release 2017.2 (geneXplain, Wolfenbüttel, Germany) (http://genexplain.com/transfac). In this study we used «Genome Enhancer» module of the pipeline «From genome to target» to identify transcription factor binding sites (TFBS) that are enriched in the promoter regions under study as compared to a background sequence set such as promoters of genes that were not differentially regulated under the condition of the experiment. We denote the study and background sets briefly as Yes and No sets. The «Genome Enhancer» module uses two algorithms for TFBS enrichment analysis: F-Match [9] and CMA (Composite Module Analyst) [16]. F-Match algorithm finds a critical value (a threshold) for the score of each PWM in the library that maximizes the Yes/No ratio under the constraint of statistical significance (see Materials and Methods). The CMA algorithm applies genetic algorithm approach to find combinations of PWMs for the co-localized sites in promoters of DEGs. Such combinations of PWMs define groups of transcription factors that bind to the promoters of these genes in synergistic manner (forming enhanceosomes [29]) and regulate expression of their target genes in very specific conditions.

**Fig. 7:**
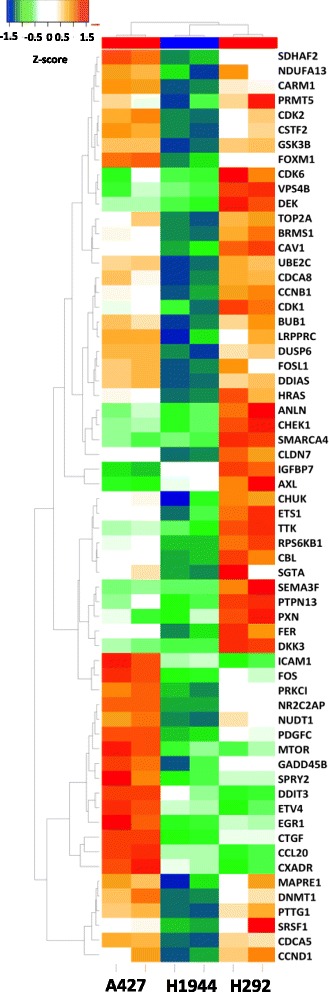
Heatmap of genes that belong to the category of “Causal Lung Neoplasms” (HumanPSD®) that were up-regulated in Nutlin-3 insensitive lung cancer cell lines (А427 and H292) in comparison to the Nutlin-3 sensitive cell line H1944. The top colored bar represents the samples with different sensitivity: red –Nutlin-3 insensitive cell lines; blue - Nutlin-3 sensitive cell line

In this work we applied these algorithms to analyze promoters of Up-regulated genes in Nutlin-3 insensitive lung cancer cell lines (А427 and H292) in comparison to the Nutlin-3 sensitive cell line H1944. We focused our attention on the up-regulated genes in order to reveal the potential resistance-specific positive feedback loops (as it is described in our previous paper [13]) that are maintained mainly through gene up-regulation. First of all, we restricted the list of up-regulated genes by FC > 1.5 (which corresponds to LogFC> 0.58). In order to characterize the up-regulated genes a bit further we mapped them to the disease ontology from the HumanPSD® databases and found a very high enrichment of genes related to different types of cancer (Additional file 5: Table S4). The most statistically significant match is the set of 118 genes to the category “Correlative Colonic Neoplasms” (*p*-value< 2.53*10^− 9^). 62 of these genes belong to the biomarkers of “Causal Lung Neoplasms” (p-value < 1.88*10^− 4^). (This gene list is present in Additional file 6: Table S5). We constructed the heatmap for these genes summarizing their expression profiles throughout all obtained microarray data (Fig. 7). One can clearly see the difference between gene expression profiles of the Nutlin-3 sensitive cell line (H1944 in the center of the heatmap) compared to the two insensitive cell lines (А427 – left side and H292 – right side of the heatmap).

Promoters were extracted from the human genome (build hg38) -1000 nucleotide upstream of TSS (start of transcription) and + 100 downstream. The result of F-Match analysis is presented in the Additional file 7: Table S6.

We also have applied the СМА (Composite Module Analyst) algorithm, which allows predicting the formation of complexes of transcription factors binding sites that could jointly regulate groups of genes, such as up-regulated in our study. As a result of application of the CMA algorithm we identified the potential complexes of TFs that may synergistically bind to promoters of these genes and maintain their elevated expression, which in turn leads to the observed relatively high resistance of these cell lines to Nutlun-3.

**Fig. 8:**
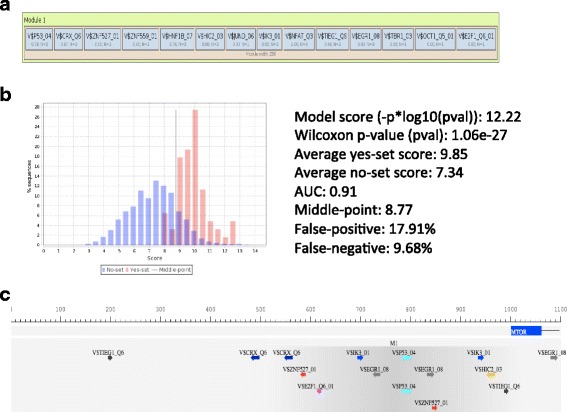
Results of CMA analysis of up-regulated genes in Nutlin-3 insensitive cell lines. **a** Combination of 14 PWMs with their optimized cut-offs identified by genetic algorithm. **b** The discriminative parameters of the composition of the Composite Score (p-value of the Wilcoxon test, AUC, rates of false positives and false negatives) and two histograms of the distributions of the Composite Score values in Yes and No promoters. C) An example of the site location in the promoter of MTOR gene. The promoter of MTOR gene contains predicted sites for p53, E2F-1, EGR1 and several other transcription factors

Results of the CMA analysis are shown in the Fig. 8 below.

We identified a combination of PWMs that discriminates the promoters of Yes and No sets rather well. This combination contains PWMs for such important cancer-related transcription factors as p53, E2F-1, EGR1, AP-1, OCT1 and others. The full list of 22 transcription factor genes encoding TFs of selected combination of PWMs is given in the Additional file 7: Table S6 (tab CMA). Many of them are associated with various Neoplasms including Lung Neoplasms.

### Finding master regulators in networks upstream of TFs

We searched for master regulator molecules in signal transduction pathways upstream of the identified transcription factors. The master-regulator search uses the TRANSPATH® database (http://genexplain.com/transpath) [17] and is implemented in the workflow “From genome to target” as a last step. The main algorithm of the master regulator search has been described earlier [13]. The goal of the algorithm is to find nodes in the global signal transduction network that may potentially regulate the activity of the set of transcription factors found at the previous step of analysis. Such nodes are considered as most potent drug targets, since any influence on such a node may switch the transcriptional programs of hundreds of genes that are regulated by the respective TFs. In our analysis we have ran the algorithm with a maximum radius of 12 steps upstream of the TFs. In order to identify the potential positive feedback loops in the system we applied additional filtering of the obtained master-regulators. We required that the Composite Score of the genes encoding master-regulators in the system were above the critical value (8.77) computed by CMA algorithm. This requirement gives possibility to find those master-regulators that regulate elevated expression of their own genes (through multiple TFBSs found in the promoters of these genes), thus leading to the feedback mechanism of maintaining its own elevated expression. In the Table 4 below we give the final list of obtained master-regulators potentially involved in maintenance of elevated resistance to Nutlin-3.

**Table 4:** List of master-regulator molecules (including complexes and modified forms) that have passed all criteria

Master molecule name	Gene symbol	logFC	Composite score	Master Regulator Score	FDR	Z-Score	Ranks sum
Cdk1(h)	CDK1	0.878	9.758	0.821	0.000	10.264	0
Cdk2(h)	CDK2	0.918	11.485	0.821	0.000	10.264	2
c-Fos(h)	FOS	0.660	9.973	0.821	0.000	10.264	4
Chk1(h)	CHEK1	0.909	9.186	0.821	0.000	10.264	6
cyclinB1(h)	CCNB1	1.380	9.670	0.821	0.000	10.264	8
mTOR(h):raptor(h)	MTOR	0.773	9.664	0.821	0.000	9.986	11
mTOR(h)	MTOR	0.773	9.664	0.821	0.000	9.986	13
Chk1(h){pS345}	CHEK1	0.909	9.186	0.821	0.000	9.707	15
GSK3beta(h){p}	GSK3B	0.937	9.181	0.821	0.000	4.971	15
E2-C(h)	UBE2C	2.270	9.919	0.821	0.000	8.593	17
p70S6K1-alpha2(h)	RPS6KB1	0.658	9.644	0.821	0.000	3.857	33
cyclinA:Cdk2{pT160}	CDK2	0.918	11.485	0.672	0.008	4.320	34
GSK3beta(h){pS9}	GSK3B	0.937	9.181	0.821	0.000	3.857	35
cyclinD:Cdk4{pT172}	CCND1	0.910	9.188	0.672	0.008	4.320	37
PKAc(h):GSK3beta(h)	GSK3B	0.937	9.181	0.821	0.000	3.578	40
cyclinD1(h):Cdk4(h)	CCND1	0.910	9.188	0.672	0.014	3.931	42
securin(h)	PTTG1	0.979	9.606	0.821	0.000	3.578	42
cyclinD1:Cdk4:PAK1	CCND1	0.910	9.188	0.672	0.014	3.905	45
Cdk4(h):cyclinD1a(h)	CCND1	0.910	9.188	0.672	0.014	3.852	47
cyclinE(h):Cdk2(h)	CDK2	0.918	11.485	0.672	0.022	3.510	56
p70S6K1(h){pT389}	RPS6KB1	0.658	9.644	0.821	0.000	2.185	89
p70S6K1(h)	RPS6KB1	0.658	9.644	0.821	0.000	1.628	146
H-Ras:GTP:Raf-1{p}	HRAS	1.381	8.501	0.589	0.041	1.490	194

**Fig. 9:**
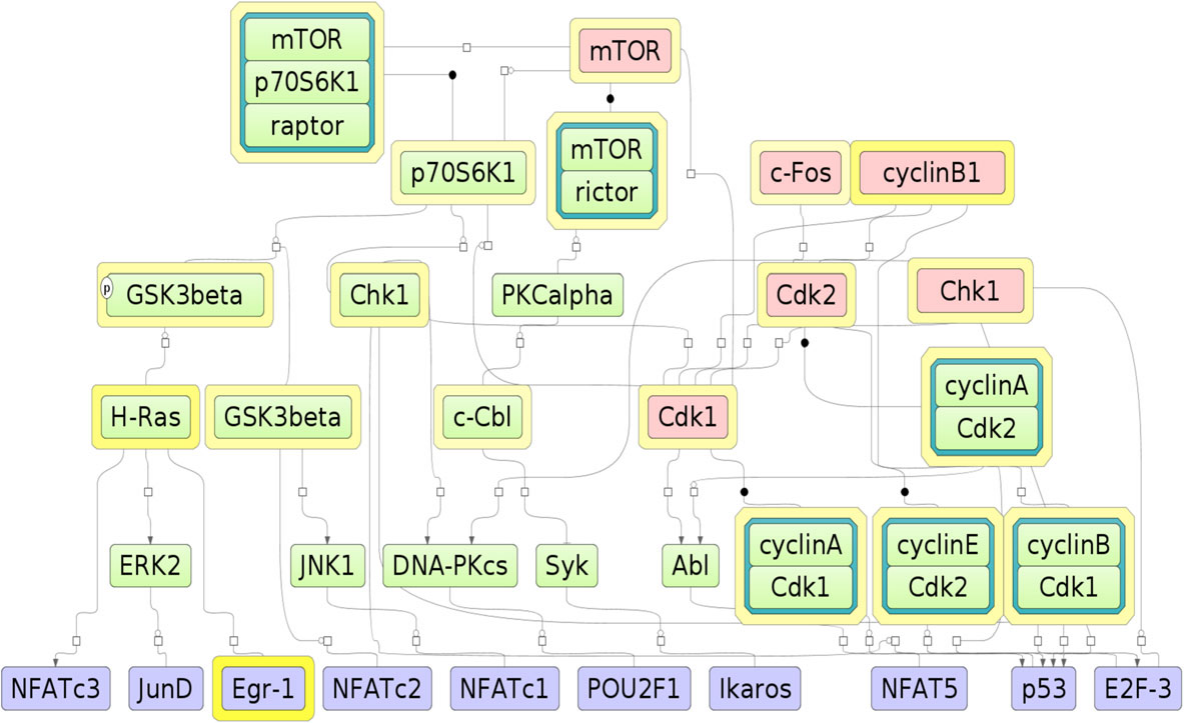
Visualization of the part of signal transduction network that connects identified master-regulators (red) with the transcription factors (blue) found in the promoter analysis of genes upregulated in Nutlin-3 insensitive cell lines. Differential expression of genes encoding the corresponding proteins on the diagram is shown by the color of the layer around the molecule. Yellow color corresponds to increased expression (up-regulation) in Nutlin-3 insensitive cell lines compared to the sensitive cell line. The intensity of the color reflects the fold change

Figure 9 bellow shows the diagram of the network constructed by the algorithm of master-regulator search. The network connects the identified top master-regulators (red) with the transcription factors found in the promoter analysis (blue).

By combining the results of gene set enrichment analysis (see above) and the master regulator search we found especially interesting that identified earlier PI3K sub-pathway (PI3K ---Mdm2-−−/ p53) involves the identified here master-regulator mTOR for inhibition of activity of p53, which is the main target of Nutlin-3 (in the complex with mdm2).

### Experimental validation by chemical inhibitors of mTOR as master-regulator

Taking all computational evidences together we choose the master-regulator mTOR as the target molecule in the context of PI3K pathway. We applied specific chemical inhibitors in order to test their effect on the survival of the selected cell lines. We choose the following chemical inhibitors (Table 5).

**Table 5:** Three chemical inhibitors used in this work for the validation of molecular targets

#	cat №	Chemical name (Cayman)	Target(s)
I1	Cay70920–5	LY294002	PI3K
I2	Cay10005229–5	2,3-DCPE (hydrochloride)	Bcl-XL
I3	Cay10565–25	NVP-BEZ235	PI3K and mTOR

To validate the effect of the inhibition of mTOR in the context of PI3K pathways we chose the dual inhibitor I3 that is known to effectively inhibit both PI3K and mTOR action. To test the mTOR-specificity of the effect we used the general inhibitor of PI3K (I1). And as a negative test we used the inhibitor of Bcl-XL (I2) that is known to be involved in cancer but was not predicted here as a master-regulator.

We use the following treatment conditions for these three inhibitors.

- I1 in concentrations: 50 μM, 25 μM, 12,5 μM, 6,25 μM, 3,1 μM and 0 μM (Control);
- I2 in concentrations: 80 μM, 40 μM, 20 μM, 10 μM, 5 μM and 0 μM (Control);
- I3 in concentrations: 50 μM, 25 μM, 12,5 μM, 6,25 μM, 3,1 μM and 0 μM (Control).

The concentration of the inhibitors was chosen according to their maximal solubility in the medium. DMSO was used to prepare the needed concentration of the inhibitors and the concentration of DMSO was the same in all tested solutions (including Control) and it was below 0.5%. Each measurement was done in three replicas. After 48 h from adding of the inhibitor we measured the percentage of the survived cells using the test with rezuverin.

We found that inhibitors I1 and I2 do not demonstrate any cytotoxic activity on all tested cell lines (data not shown). Under any tested concentrations of the inhibitors the number of the survived cells were the same as in the Control and did not differ significantly from 100%.

**Fig. 10:**
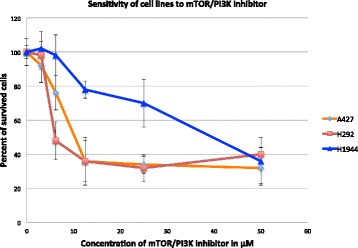
Graphics of the viability test of the three lung cancer cell lines to compound NVP-BEZ235 – the dual inhibitor of PI3K and mTOR. The colored lines represent effects of increasing concentrations of the inhibitors on the survival of three cell lines: sensitive to Nutlin-3 (H1944) and insensitive to Nutlin-3 cell lines (А427 and H292)

In contrast, the inhibitor I3 demonstrated a moderate cytotoxic activity with the following IC50 values in tested cell lines: A427 (IC50 = 11.8 ± 3.4), H292 (IC50 = 6.05 ± 2.1), DV-90 (IC50 = 19.9 ± 4.6), Н1944 (IC50 = 34.9 ± 3.6) и Н2228 (IC50 = 27.1 ± 6.6). For the three cell lines of our interest the dose-effect curves are shown in Fig. 10 below.

We found that Nutlin-3 insensitive cell lines (A427 and H292) exhibit the highest sensitivity to the dual chemical inhibitor of mTOR-PI3K whereas the Nutlin-3 sensitive cell line (H1944) appeared to be relatively insensitive to this inhibitor. These results confirmed our prediction of the master regulator mTOR in the PI3K signaling pathway, which, most probably, is responsible for the low sensitivity of particular lung cancer cell lines to treatment by the p53-reactivating compound Nutlin-3.

## Discussion

Resistance and low sensitivity to chemotherapy and targeted therapy of the cancer cells is one of the biggest problems of cancer treatment. In this work we studied the molecular mechanisms of low sensitivity of cancer cells to the p53-reactivating compound Nutlin-3 using genome-wide transcriptomics profiling followed by causative computational analysis. In order to analyze the effect of Nutlin-3 on lung cancer cells and understand the mechanisms of low sensitivity of some of them to the treatment, we performed an extensive study of the biological activity of the compound Nutlin-3 on a number of lung cancer cell lines. We pay especial attention to the lung cancer cell lines that although caring wild-type TP53 gene (which, in the complex with Mdm2, is the primary target of Nutlin-3) are still very different in their sensitivity to Nutlin-3 treatment. We identified that the cell line H1944 is the most sensitive to the treatment by Nutlin-3. Even under the lowest concentration of the compound the cells of this cell line were rapidly dying. Other cell lines, such as A427 and H292 showed relatively low sensitivity to the treatment by Nutlin-3. The death of cells was triggered only under relatively high concentration of the compound. It is interesting to see that both cell lines A427 as well as NCI-H292 demonstrated rather high sensitivity to another p52-Mdm2 inhibitor AMGMDS3 (IC_50_ = 0.49 μM for H292 and 0.55 μM for A427) [30]. So, it seems that these two inhibitors, although very similar in their main target are rather different in their overall effect on signaling pathways in the cancer cells. We compared the gene expression profiles of these cell lines before and after treatment by Nutlin-3 and identified several hundreds of genes whose expression was significantly different between sensitive and the insensitive cell lines. We identified 623 DEGs before the treatment, 674 DEGs after the treatment by 5 μM of Nutlin-3 and 626 DEGs after the treatment by 30 μM of Nutlin-3. The GSEA analysis of pathway enrichment by these genes gave us the first clue about the main processes involved in regulation of insensitivity to Nutlin-3. Among the most enriched pathways were p53 pathway, E2F network, Aurora-B and A, cell cycle regulation pathway and other pathways involved in regulation of different processes related to regulation of cell cycle. It is known that the cell line NCI-H292, the one among two of the cell lines that we identified as relatively insensitive to Nutlin-3, contains the CRTC1-MSML2 gene fusion [31], which is quite common in the mucoepidermoid carcinomas (a subtype of the lung cancer of the NCI-H292 cell line). We were interested if the relative insensitivity of H292 cell line can be explained by presence of this gene fusion. We compared the differentially expressed genes revealed in our study with the genes identified as regulated by the CRTC1-MSML2 fusion, that were revealed in an extensive microarray study upon knockdown of the chimeric transcription regulator that is expressed in in human mucoepidermoid carcinoma cells as the result of the CRTC1-MSML2 fusion [31]. We found a very small overlap between these gene lists. Among 623 genes differentially expressed in Nutlin-3 insensitive cell lines and 641 genes up- or down-regulated upon CRTC1-MSML2 fusion knockdown we can see only 18 overlapping genes, which is statistically insignificant overlap. Out of these 18 genes 11 genes were belong to the rather general GO category “negative regulation of cellular process” (*p*-value < 10^− 5^). Therefore we found no evidence of the influence of CRTC1-MSML2 fusion on the resistance mechanism to Nutlin-3. It seems that the molecular pathways that are involved in oncogenic program maintained in such cancer subtypes through the CRTC1-MSML2 fusion are rather different from the pathways maintaining the resistance of these cells to the cytotoxic effect of Nutlin-3.

In order to identify such pathways we searched for potential master regulators applying our upstream analysis approach. From the perspective of searching for such master regulators we focus our attention first of all on the genes that have higher expression values in the insensitive cell lines compared to the sensitive (up-regulated genes). Analysis of promoters of these genes helped us to identify several transcription factors with enriched binding sites found in co-localized clusters. We believe that such clusters reflect position and composition of very specific enhanceosome that is formed at the promoters of up-regulated genes and controls their elevated expression in the insensitive cell line. On the next step of our study we analyzed signal transduction network upstream of the revealed transcription factors in order to understand the potential molecular mechanism of activation of these transcription factors. The goal of such analysis was to find few master-regulators in this network that might exert their control on the transcription factors found on the first step.

Finally, after performing the search for potential master regulators, we checked which of them were actually up-regulated by themselves. So, we require that genes, which are expressing proteins that were found by the algorithm as potential master regulators, should have significantly higher expression in the insensitive cells compared to the sensitive cells. This reflects the presence of positive feedback loop in the system. We hypothesized that the observed increase of resistance might be supported by the presence of positive feedback loops. We can observe such loops in the network when the genes, expressing master-regulator proteins, are working under control of the transcription factors, which receive activating signals through the signaling cascade, containing proteins, which are expressed by these genes (master regulators). Therefore, the up-regulation of the genes, encoding master regulators in this analysis, indicates the presence of such feedback loops. We think that such positive feedback loops can contribute to the stabilization of the resistance to Nutlin-3 (and potentially to other anticancer compounds with similar mechanisms of action, such as p53 reactivator molecule RITA, which was studied in our previous work [32]), since they maintain activation of a certain set of critically important genes through the auto-activation loop.

As a result, we revealed the following master regulator genes: MTOR, CDK1, CDK2, CHK1, cyclin-B1. We noticed that many of the suggested master regulators are very important proteins that are known to be involved in regulating such processes as cell cycle and apoptosis.

To answer the question of how commonly the mTOR, cell cycle and PI3K pathways are involved in regulation of sensitivity to anti-cancer compounds with similar mechanism of action as Nutlin-3 we intersected the data on sensitivity to the MDM2 inhibitor (AMGMDS3 compound) described in the paper Saiki et al. [30] with the biggest resource of gene expression data on various cancer cell lines – the CCLE (Cancer Cell Line Encyclopedia) [33, 34]. We were interested in the cell lines that are characterized by wild-type p53. We identified only very few data sets of lung cancer cell lines that have both AMGMDS3 sensitivity data and gene expression data in CCLE (namely, cell lines A427, A549, NCI-H292, NCI-H460, that actually were characterized by very similar AMGMDS3 IC50 values - all were quite sensitive to this particular inhibitor). Therefore, we decided to analyse all available p53wt cell lines of all types of cancer that have got both gene expression data in CCLE and sensitivity data to the MDM2 inhibitor. We identified 52 of such cell lines with sensitivity (IC50) to the AMGMDS3 compound varying from 0.0074 to 50 μM. We identified genes whose expression in different cell lines correlate with IC50 values. We found 168 genes positively correlated with IC50 (insensitivity to the Mdm2 inhibitor) and 227 genes negatively correlated (*p*-value < 0.01) (Additional file 8: Table S7). Pathway analysis of this gene expression correlations using GSEA method reveals PI3K pathway as one of the most important survival mechanism of these cell lines against AMGMDS3 compound (Additional file 9: Table S8). This coincides partially with the results of the analysis of Nutlin-3 sensitivity that is done in our current study. Here we have shown that the PI3K pathway provides a context for the mTOR to play as a potential mater regulator. Nevertheless, in the analysis of the 52 cell lines we did not identify mTOR pathway. This demonstrates that there is certain specificity in the mechanism of decreased sensitivity to Nutlin-3 of two lung cancer cell lines analysed in our study as compared to the sensitivity to the AMGMDS3 compound.

Taking all this into account, we performed experimental validation of the computational predictions. We found that Nutlin-3 insensitive cell lines (A427 and H292), in turn, exhibit the highest sensitivity to the dual chemical inhibitor of mTOR-PI3K, whereas the Nutlin-3 sensitive cell line (H1944) appeared to be relatively insensitive to this inhibitor.

## Conclusions

The results of this work confirmed our prediction of the master regulators in the mTOR-PI3K signaling pathway responsible for the low sensitivity of these two particular lung cancer cell lines to treatment by the p53-reactivating compound Nutlin-3. As we predicted, the Nutlin-3 insensitive cell lines appeared to be highly sensitive to the inhibitors of mTOR-PI3K pathway. These findings suggest potential preclinical in vitro study to evaluate combining MDM2-TP53 interaction inhibitor(s) with mTOR-PI3K inhibitor(s) across large panel of TP53 WT cell lines using clinically relevant concentrations of clinically relevant inhibitors.

## Additional files

Additional file 1: Figure S1. R-script for Limma calculation. It includes options for blocks of correlated measurements (technical replicates). **Figure S2.** Comparison of genes belonging to different TRASPATH® pathways. Sixteen overlapping genes are the genes encoding proteasome subunits. PISK pathway is most different from other 3 related pathways of cell cycle control. **Figure S3.** A diagram of top 9 gene promoters (out of 62) with the results of CMA analysis. Exons are shown as blue boxes. The TF binding sites identified by CMA are shown as colored arrows. Gray background shows the position of the site cluster in the promoter. (DOCX 154 kb)

Additional file 2: Table S1. Normalized expression values of all genes with detected expression in the studies conditions and mapped to Ensembl. In the tab “Nsen vs Sen” we give the results of Limma analysis of the LogFC between Nutlin-3 insensitive (Nsen) and sensitive cell lines. (XLSX 3291 kb)

Additional file 3: Table S2. GO analysis of all 7 sets of genes - Up- and Down- regulated genes upon treatment by Nutlin-3 in two concentrations 5 μM and 30 μM of Nutlin-3 (*p*-value< 0.05, LogFC> 0.58 (which corresponds to FC > 1.5) for up-regulated and LogFC<− 0.58 for down-regulated genes). Parameter Sum_Logpval sums up logarithms of *p*-values for one GO term in different conditions of treatment. It allows to sort GO terms according to their total significance in all conditions. (XLSX 334 kb)

Additional file 4: Table S3. Results of gene set enrichment analysis (GSEA) of the obtained three gene expression profiles of differences between sensitive and insensitive lung cancer cell lines. For that we used geneXplain platform and applied the pathways ontology of TRANSPATH® database. (XLSX 147 kb)

Additional file 5: Table S4. Results of mapping of upregulated genes on the HumanPSD® disease ontology for all three conditions of Nutlin-3 treatment. The results contain the number of matched genes with the respective disease the calculated p-value and adjusted p-value of such match. (XLSX 78 kb)

Additional file 6: Table S5. List of 62 genes up-regulated in Nutlin-3 insensitive cell lines and matching the disease category “Causal Lung Neoplasms”. This list is used for the promoter analysis. (XLSX 61 kb)

Additional file 7: Table S6. Result of F-Match analysis of the promoters of 62 genes up-regulated in Nutlin-3 insensitive cell lines. We show the list of transcription factors whose sites were detected as overrepresented in the promoters. We also show the respective PWMs linked to these transcription factors, the Yes/No ration of the site frequency and the LogFC for the expression of the transcription factor gene. In Tab CMA we give results of CMA analysis. 22 TF were revealed. (XLSX 59 kb)

Additional file 8: Table S7. Results of correlation analysis of gene expression in 52 cancer cell lines and their sensitivity (IC50) value towards Mdm2 inhibitor AMGMDS3. We found 168 genes positively correlated with IC50 (insensitivity to the Mdm2 inhibitor) and 227 genes negatively correlated (p-value < 0.01). (XLSX 2438 kb)

Additional file 9: Table S8. Pathway analysis of the gene expression correlations using GSEA method and TRANSPATH pathway ontology. (XLSX 103 kb)
