# Supplementary material for: Computational master-regulator search reveals mTOR and PI3K pathways responsible for low sensitivity of NCI-H292 and A427 lung cancer cell lines to cytotoxic action of p53 activator Nutlin-3
Source: BMC Med Genomics. 2018 Feb 13;11(Suppl 1):12. doi: 10.1186/s12920-018-0330-5 (PMC5836833; doi:10.1186/s12920-018-0330-5)
Supplement: Supplementary file 1 — R-script for Limma calculation. It includes options for blocks of correlated measurements (technical replicates). Figure S2. Comparison of genes belonging to different TRASPATH® pathways. Sixteen overlapping genes are the genes encoding proteasome subunits. PISK pathway is most different from other 3 related pathways of cell cycle control. Figure S3. A diagram of top 9 gene promoters (out of 62) with the results of CMA analysis. Exons are shown as blue boxes. The TF binding sites identified by CMA are shown as colored arrows. Gray background shows the position of the site cluster in the promoter. (DOCX 154 kb) [file 12920_2018_330_MOESM1_ESM.docx]

designf <- model.matrix(~0+factor(c(1,1,2,2,3,3,4,4,5,5,6,6,1,1,2,2,3,3)))

colnames(designf) <- c("Res_N30","Res_N5","Res_Co","Sen_N30","Sen_N5","Sen_Co")

biolrepf <- c(1,1,2,2,3,3,4,4,5,5,6,6,7,7,8,8,9,9)

corfitf <- duplicateCorrelation(datalog2, ndups=1, block= biolrepf)

fitf <- lmFit(datalog2,designf,block = biolrepf, cor = corfitf$consensus)

contrast.matrixf <-makeContrasts(Res_Co-Sen_Co, Res_N5-Sen_N5, Res_N30-Sen_N30, Res_N5-Res_Co, Sen_N5-Sen_Co, Res_N30-Res_Co, Sen_N30-Sen_Co, levels=designf)

fitf2 <- contrasts.fit ( fitf, contrast.matrixf)

fitf2B <- eBayes(fitf2)

toptable(fitf2B, number=20, coef=1)

> f1 <- toptable(fitf2B, coef=1, number=Inf,resort.by="logFC")

> f2 <- toptable(fitf2B, coef=2, number=Inf,resort.by="logFC")

> f3 <- toptable(fitf2B, coef=3, number=Inf,resort.by="logFC")

> f4 <- toptable(fitf2B, coef=4, number=Inf,resort.by="logFC")

> f5 <- toptable(fitf2B, coef=5, number=Inf,resort.by="logFC")

> f6 <- toptable(fitf2B, coef=6, number=Inf,resort.by="logFC")

> f7 <- toptable(fitf2B, coef=7, number=Inf,resort.by="logFC")

Figure FS1. R-script for Limma calculation. It includes options for blocks of correlated measurements (technical replicates).


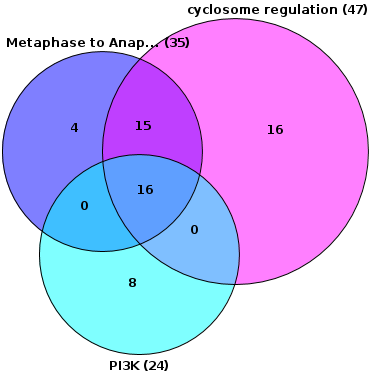

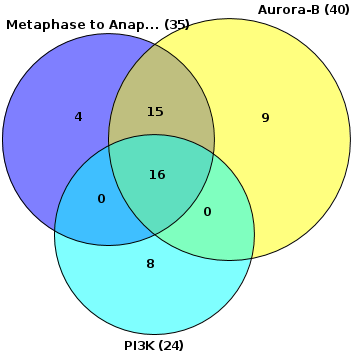

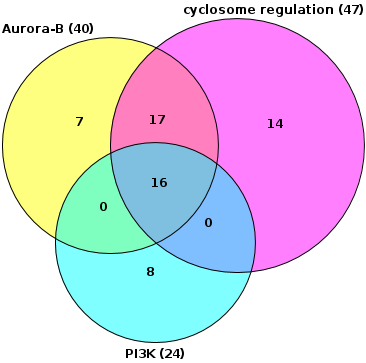


Figure FS2. Comparison of genes belonging to different TRASPATH® pathways. 16 overlapping genes are the genes encoding proteasome subunits. PISK pathway is most different from other 3 related pathways of cell cycle control.


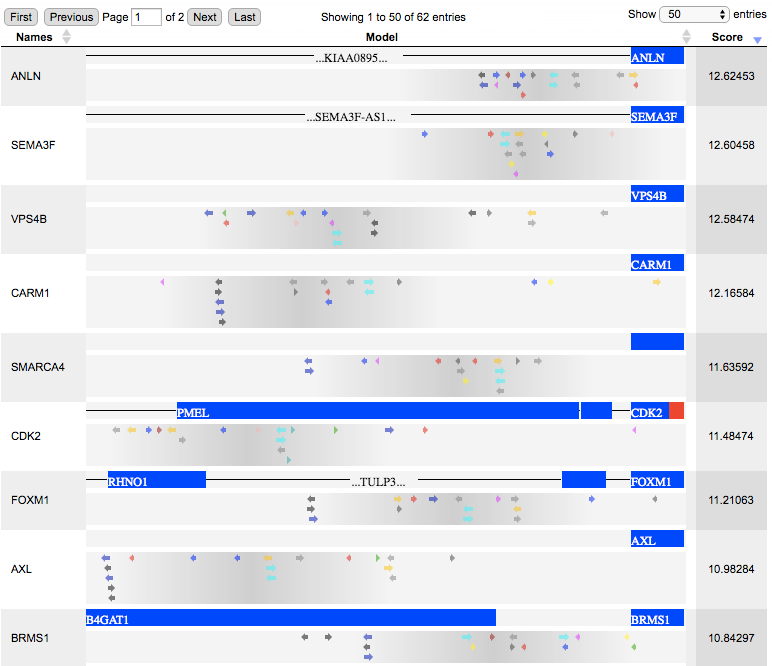


Figure FS3. A diagram of top 9 gene promoters (out of 62) with the results of CMA analysis. Exons are shown as blue boxes. The TF binding sites identified by CMA are shown as colored arrows. Gray background shows the position of the site cluster in the promoter.
